# Supplementary material for: The maternal brain is more flexible and responsive at rest: effective connectivity of the parental caregiving network in postpartum mothers
Source: Sci Rep. 2023 Mar 23;13:4719. doi: 10.1038/s41598-023-31696-4 (PMC10036465; doi:10.1038/s41598-023-31696-4)
Supplement: Supplementary file 1 — Supplementary Information. [file 41598_2023_31696_MOESM1_ESM.pdf]

The maternal brain is more flexible and responsive at rest: effective connectivity of the parental caregiving network in postpartum mothers

Orchard, Voigt, Chopra, Thapa, Ward, Egan, Jamadar

~ Supplementary Information ~

1. Spectral Dynamic Causal Modelling

Dynamic causal modelling (DCM) is a Bayesian framework that allows directed (causal) connections to be inferred between neural systems: known as *effective connectivity*. In resting-state data, the DCM approach is based on a deterministic model that generates cross-spectra predictions, and is referred to as *spectral DCM* (spDCM)[1]. We have previously applied spDCM to studies of resting-state connectivity in obesity[2] and decision-making[3]. Full details of the spDCM approach is provided in Friston et al.[1] and Voigt et al. [2], but is described briefly here.

In order to model brain activity in the absence of external stimuli or evoked responses, the spDCM model adds a stochastic component to the classic DCM based on ordinary differential equations. Mathematically, the stochastic generative model is expressed using two equations:

The neuronal state equation (S1):

$$\dot{x}(t) = f(x(t), u(t), \theta) + v(t) \qquad \text{(S1)}$$

And the observation equation (S2), a static nonlinear mapping from the hidden physiological states in (S1) to the observed BOLD activity:

$$y(t) = h(x(t), \varphi) + e(t), \qquad \text{(S2)}$$

where $\dot{x}(t)$ is the rate of change of the neuronal states x(t), θ are unknown parameters (i.e., the effective connectivity) and v(t) (resp. e(t)) is the stochastic process – called the state noise (resp. the measurement or observation noise) – modelling the random neuronal fluctuations that drive the resting state activity. In the observation equations, φ are the unknown parameters of the (haemodynamic) observation function and u(t) represents any exogenous (or experimental) inputs that drive the hidden states – that are usually absent in resting state designs. spDCM furnishes a constrained inversion of the stochastic model by parameterising the neuronal fluctuations $v(t)$. spDCM simplifies the generative model by replacing the original timeseries with its second-order statistic, the cross-spectra. As such, instead of estimating time-variant hidden states, spDCM estimates their temporally-invariant covariance.

The parametric empirical Bayes (PEB) framework[4] was used to test for differences in effective connectivity. Empirical Bayes refers to the process of Bayesian model inversion or fitting of hierarchical models. In a hierarchical model, the constraints on the posterior density of model parameters at a given level is provided by the level above. The constraints are called empirical priors because they are informed by the data. PEB is a second-level model over parameters, and represents how individual within-subject connections derive from the subjects' group membership. PEB relies upon Bayesian Model Reduction (BMR) to refine the inversion of multiple models of a single dataset, or a single hierarchical model of multiple datasets. BMR can improve

subject-specific parameter estimates by using group-level estimates to inform the calculation of individual DCMs from local optima.

2. Effect of Wellbeing

In general, the effect of wellbeing was modest across all analyses (Supplementary Figure 1). For the main effect of group, no additional connections were identified when controlling for wellbeing (Supplementary Table 1). In the cognition analysis, when controlling for the effect of wellbeing, mothers with better cognitive performance show increased self-inhibition of the parahippocampal gyrus, but no longer show the bottom-up inhibition from the nucleus accumbens to the dorsomedial prefrontal cortex (Supplementary Table 2). For social cognition, mothers with better social cognition also showed decreased top-down inhibition of the nucleus accumbens from the dorsomedial prefrontal cortex when controlling for the effect of wellbeing (Supplementary Table 3). Finally, for maternal caregiving, mothers with better maternal caregiving also showed increased inhibition of the amygdala from the nucleus accumbens, and decreased top-down inhibition of the amygdala from the dorsomedial prefrontal cortex when controlling for the effect of wellbeing (Supplementary Table 4).

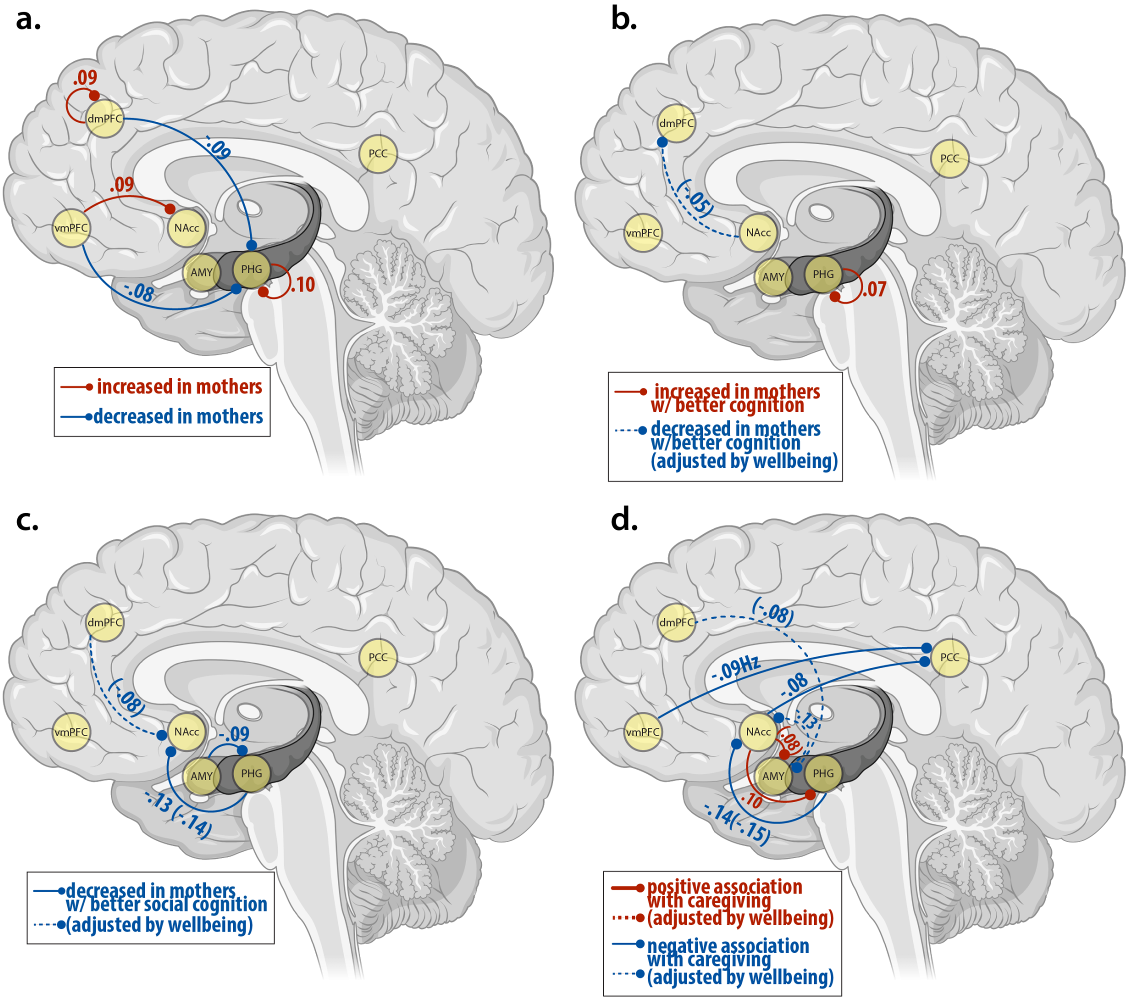

Supplementary Figure 1: *Schematic diagram showing spDCM results for (a.) the main effect of group (mothers vs non-mothers), (b.) the interaction effect of group-by-cognition, (c.) the interaction effect of group-by-social cognition, and (d.) the main effect of maternal caregiving in mothers only. All connections are inhibitory. Values represent effect sizes (see Table 2-5) in Hz. Blue lines depict decreased inhibition and red lines depict increased inhibition. Dashed lines represent connections that are attenuated when the model adjusts for wellbeing, and solid lines are connections which are present whether or not wellbeing is adjusted for. Abbreviations: PCC, posterior cingulate cortex; vmPFC, ventromedial prefrontal cortex; dmPFC, dorsomedial prefrontal cortex; PHG, parahippocampal gyrus; Amyg, amygdala; NAcc, nucleus accumbens.*

Supplementary Table 1: *Effect size, valence and posterior probabilities for each connection showing a main effect of group (mothers>non-mothers)*

Connection	Valence	*Mothers vs. Non-Mothers*		*Mothers vs. Non-Mothers (controlled by wellbeing)*	
		Effect Size in Hz [95% CI]	Posterior Probability	Effect Size in Hz [95% CI]	Posterior Probability
dmPFC→ dmPFC	Inhibition	0.09 [0,0.16]	0.97	0.08 [0, 0.16]	0.96
dmPFC → PHG	Inhibition	-0.09 [-0.19,0]	0.97	-0.10 [-0.19, -0.02]	0.98
vmPFC → PHG	Inhibition	-0.08 [-0.16,0]	096	-0.08 [-0.16, 0]	0.95
vmPFC → NAcc	Inhibition	0.09 [0.01,0.17	0.96	0.09 [0.01, 0.17]	0.96
PHG → PHG	Inhibition	0.10 [0.18,0.01]	0.98	0.11 [0.03, 0.19]	0.99

Abbreviations: Hertz (Hz), Confidence Interval (CI), dorsomedial prefrontal cortex (dmPFC), ventromedial prefrontal cortex (vmPFC), parahippocampal gyrus (PHG), amygdala (Amyg) nucleus accumbens (NAcc).

Supplementary Table 2: *Effect size, valence and posterior probabilities for each connection showing an interaction effect of group-by-cognition, with and without controlling for wellbeing*

Connection	Valence	*Group-by-Cognition*		*Group-by-Cognition (controlling for Wellbeing)*	
		Effect Size in Hz [95% CI]	Posterior Probability	Effect Size in Hz [95% CI]	Posterior Probability
PHG → PHG	Inhibition	0.07 [0.01, 0.12]	0.98	0.07 [0.015, 0.126]	0.98
NAcc → dmPFC	Inhibition	-0.05 [-0.11, -0.00]	0.95		

Abbreviations: Hertz (Hz), Confidence Interval (CI), dorsomedial prefrontal cortex (dmPFC), parahippocampal gyrus (PHG), nucleus accumbens (NAcc).

Supplementary Table 3: *Effect size, valence and posterior probabilities for each connection showing an interaction effect of group-by-social cognition, with and without controlling for wellbeing*

Connection	Valence	*Group-by-Social Cognition*		*Group-by-Social Cognition (controlling for Wellbeing)*	
		Effect Size in Hz [95% CI]	Posterior Probability	Effect Size in Hz [95% CI]	Posterior Probability
PHG → NAcc	Inhibition	-0.13 [-0.21, -0.05]	1.00	-0.14 [-0.22, -0.05]	1.00
Amyg → PHG	Inhibition	-0.09 [-0.18, -0.01]	0.97	-0.09 [-0.18, -0.01]	0.97
dmPFC → NAcc	Inhibition			-0.08 [-0.16, -0.00]	0.95

Abbreviations: Hertz (Hz), Confidence Interval (CI), dorsomedial prefrontal cortex (dmPFC), ventromedial prefrontal cortex (vmPFC), parahippocampal gyrus (PHG), amygdala (Amyg) nucleus accumbens (NAcc).

Supplementary Table 4: *Main effect of maternal caregiving, with and without controlling for wellbeing*

Connection	Valence	*Maternal Caregiving*		*Maternal Caregiving (controlling for Wellbeing)*	
		Effect Size in Hz [95% CI]	Posterior Probability	Effect Size in Hz [95% CI]	Posterior Probability
vmPFC → PCC	Inhibition	-0.09 [-0.17, -0.01]	0.97	-0.09 [-0.02, -0.02]	0.98
PHG → NAcc	Inhibition	-0.14 [-0.22, -0.06]	1.00	-0.15 [-0.22, -0.07]	1.00
Amyg → NAcc	Inhibition	-0.13 [-0.20, -0.05]	1.00	-0.13 [-0.20, -0.05]	1.00
NAcc → PCC	Inhibition	-0.08 [-0.16, -0.01]	0.97	-0.08 [-0.16, -0.00]	0.96
NAcc → PHG	Inhibition	0.1 [0.02, 0.18]	0.98	0.1 [0.03, 0.18]	0.99
dmPFC → Amyg	Inhibition			-0.08 [-0.16, -0.00]	0.96
NAcc → Amyg	Inhibition			0.08 [0.00, 0.15]	0.95

Abbreviations: Hertz (Hz), Confidence Interval (CI), dorsomedial prefrontal cortex (dmPFC), ventromedial prefrontal cortex (vmPFC), parahippocampal gyrus (PHG), posterior cingulate cortex (PCC), amygdala (Amyg), nucleus accumbens (NAcc).

3. Cognition Summary Scores

Supplementary Tables 5-8 report the principle components results for the cognition data. Supplementary Table 9 reports the means and standard deviations of the cognitive data. Please refer to Orchard et al. (2021, Journal of Women's Health) for full report and discussion of the cognitive results. Note that the values are slightly different here compared to our previous report, as some data was excluded for fMRI data quality (see Methods). However, all effects show the same trends as reported in Orchard et al. (2021)

Supplementary Table 5: *Cognitive Performance Principal Component Coefficient Loadings, Variance Explained, and Eigenvalues for each component.*

	PC1	PC2	PC3	PC4
Hopkins Verbal Learning Test	0.48	0.48	0.73	-0.06
Digit Span Forwards	0.54	-0.43	-0.02	0.72
Digit Span Backwards	0.53	-0.49	-0.07	-0.69
Symbol Digit Modalities Task	0.44	0.59	-0.68	-0.00
Variance Explained (%)	54.59	23.18	13.32	8.91
Eigenvalue	**2.18**	0.93	0.53	0.36

Supplementary Table 6: *Social Cognition Principal Component Coefficient Loadings, Variance Explained, and Eigenvalues for each component.*

	PC1	PC2
Reading the Mind in the Films Task	0.71	0.71
Toronto Empathy Questionnaire	0.71	-0.71
Variance Explained (%)	51.20	46.76
Eigenvalue	**1.02**	0.94

Supplementary Table 7: *Maternal Caregiving Principal Component Coefficient Loadings, Variance Explained, and Eigenvalues for each component.*

	PC1	PC2
Maternal Postnatal Attachment Questionnaire	0.71	-0.71
Maternal Self-Efficacy Questionnaire	0.71	0.71
Variance Explained (%)	84.22	16.81
Eigenvalue	**1.68**	0.34

Supplementary Table 8: *Wellbeing Principal Component Coefficient Loadings, Variance Explained, and Eigenvalues for each component.*

	PC1	PC2	PC3
Pittsburgh Sleep Quality Index	0.55	0.82	-0.16
Beck Depression Inventory	0.60	-0.26	0.76
Beck Anxiety Inventory	0.59	-0.51	-0.63
Variance Explained (%)	66.73	19.25	14.02
Eigenvalue	**2.00**	0.58	0.42

Supplementary Table 9: *Means and standard deviations of tasks and questionnaires which comprise the cognitive performance, social cognition, and wellbeing principal components.* α*=.05/9 = .0056*

	**Mean (SD)** Mothers	**Mean (SD)** Non-Mothers	***t*-value**	**Cohen's *d***	***p*-value**
Cognitive Performance					
Hopkins Verbal Learning Test	31.18 (3.1)	30.64 (3.8)	0.68	0.15	.50
Digit Span Forward	7.03 (1.2)	7.59 (1.2)	-2.10	-0.47	.039
Digit Span Backward	6.43 (1.0)	6.87 (1.4)	-1.66	-0.38	.098
Symbol Digit Modalities Task	58.73 (7.3)	61.64 (9.9)	-1.50	-0.34	.14
Social Cognition					
Reading the Mind in the Films Task	66.59 (9.2)	63.17 (11.1)	1.49	0.34	.14
Toronto Empathy Questionnaire	50.18 (4.8)	46.70 (4.2)	3.45	0.78	<.001
Wellbeing					
Pittsburgh Sleep Quality Index	7.33 (3.1)	4.67 (2.6)	4.17	0.94	<.001
Beck Depression Inventory	8.55 (5.6)	4.95 (4.3)	3.20	0.72	.002
Beck Anxiety Inventory	10.70 (8.6)	8.56 (6.63)	1.30	0.29	.20

4. Correlation Between Cognitive, Social Cognitive, Caregiving and Wellbeing Measures with Time Since Postpartum (Age of Infant)

Supplementary Table 10: Correlations between time postpartum (age of child) and each of the cognitive, social cognition, maternal caregiving, and wellbeing scores analysed.

	r-value	*p*-value
Cognitive Performance		
Hopkins Verbal Learning Test	0.06	.72
Digit Span Forward	-0.20	.21
Digit Span Backward	-0.04	.81
Symbol Digit Modalities Task	0.02	.90
Social Cognition		
Reading the Mind in the Films Task	0.24	.13
Toronto Empathy Questionnaire	-0.02	.90
Maternal Caregiving		
Attachment	-0.25	.13
Self-Efficacy	-0.17	.31
Wellbeing		
Pittsburgh Sleep Quality Index	0.19	.47
Beck Depression Inventory	0.07	.65
Beck Anxiety Inventory	-0.03	.83

N.B. Values are reported uncorrected for multiple comparisons.

5. Nucleus Accumbens and Parahippocampal Gyrus: Hub Regions of the Maternal Brain

The results indicated that the nucleus accumbens and the parahippocampal gyrus are 'hub' regions of the maternal caregiving brain network. Both regions showed significant connections in every model tested: main effect of group, main effect of maternal caregiving, interaction effect of group-by-cognition, and interaction effect of group-by-social cognition. Both the nucleus accumbens and parahippocampal gyrus are part of both the ventromedial emotion regulation system[5] and the affective limbic network[6], suggesting that the increased influence of these regions supports, and perhaps integrates, the functions of both networks.

The nucleus accumbens, part of the striatum and mesolimbic reward circuit, is considered a crucial region in the human maternal brain[7], showing both structural and functional neural adaptations across the peripartum period. The nucleus accumbens decreases in volume up to 25% across pregnancy[8], and at two-months postpartum[9], with greater volume reductions associated with increased activation in response to infant stimuli[10]. The striatum also shows grey matter volume increases later in the postpartum period. Specifically, consistent increases in grey matter volume were found in the caudate[11,12], putamen[11], nucleus accumbens[13], and pallidum[11]. Studies of resting state functional connectivity have also highlighted the role of the nucleus accumbens in sensitive maternal caregiving behaviours[14,15]. Increased connectivity between the nucleus accumbens and the amygdala is associated with a mother's ability to scaffold appropriate interactions with her child[14]. Positive maternal caregiving behaviours were also related to functional connectivity between the nucleus accumbens and the dorsomedial prefrontal cortex[15], and activation of the nucleus accumbens is correlated with increased oxytocin levels[15]. Our results, alongside the extant literature highlight the

importance of the nucleus accumbens in the global functioning of the maternal brain. Our results emphasise the influence of the nucleus accumbens for maternal cognition, and social cognition, and that the increased inhibitory influence of the nucleus accumbens may play a central role in maternal caregiving.

The parahippocampal gyrus also undergoes structural plasticity in motherhood, with grey matter changes across pregnancy[8], the postpartum period[11], and in late-life[16]. The parahippocampal gyrus is involved in memory consolidation and cohesion[17] and mediates cortico-hippocampal communication[18]. Altered parahippocampal structure and function is potentially related to changes in maternal cognition across pregnancy[19]. However, the involvement of the parahippocampal gyrus appears to extend beyond the cognitive domain. The parahippocampal gyrus is implicated across many studies of maternal brain function and sensitive caregiving[8,11,16], suggesting this region has a broader role in maternal caregiving. A recent meta-analysis has highlighted the parahippocampal gyrus as one of only six brain regions that show consistent activation in response to visual stimuli of a mother's own child, compared to an unknown control child[20]. Studies of resting state functional connectivity show disrupted connectivity between the parahippocampal gyrus, and the posterior cingulate cortex[21], amygdala[21], and dorsomedial prefrontal cortex[22]. Taken together, the parahippocampal gyrus is a key region of the maternal brain, which deserves increased attention in future studies.

6. Social Cognition

Compared to non-mothers, mothers had significantly higher scores on measures of social cognition (theory of mind, and empathy; Supplementary Tables 1; 5). Social cognition abilities contribute to successful social interactions and are important for human survival and successful maternal behaviours[23]. Motherhood requires a heightened focus on infant-related responsibilities, and for a mother to be more in tune with the thoughts, feelings, and needs of their children, especially pre-verbal infants, where infant needs must be interpreted without verbal communication[24]. This result is consistent with superior social cognition found in pregnant women, where mothers show improved ability to encode emotional faces during late pregnancy, compared to early pregnancy[25], and increased facial recognition compared to non-pregnant women[26]. Interestingly, both the Reading the Mind in the Films Task and the Toronto Empathy Questionnaire are designed to assess social cognition in adults, are not specific to motherhood or the peripartum period, and do not contain questions or items about caring for children[27,28]. These observations support the interpretation that mothers' superior social cognition performance may generalise beyond the context of childcare-related skills and behaviours, to abilities that may benefit mothers in their social interactions with other adults.

7. Selective Modulation of Inhibitory Connectivity in Motherhood

Our findings suggest a selective modulation of inhibitory pathways in motherhood. Inhibition in a cortical hierarchy serves to maintain efficient neural signalling by modulation of the primary inhibitory neurotransmitter, gamma-aminobutyric acid (GABA)[40,68]. In pregnancy and the postpartum period, GABAergic activation is modulated by fluctuating concentrations of neurosteroids, such as allopregnanolone

(a metabolite of progesterone). Allopregnanolone acts at the GABA-A receptor to increase inhibition[69,70], and GABA-A receptor density and sensitivity are flexibly upregulated and downregulated to maintain an ideal homeostatic level of inhibition[68,69] (i.e., at times of high allopregnanolone, receptor density and sensitivity are down-regulated, and vice versa). Allopregnanolone concentrations fluctuate across the peripartum period, with high levels in late pregnancy, followed by a rapid decline at parturition. Allopregnanolone levels remain low throughout the postpartum period until the return of menstruation, and cessation of breastfeeding. Weaning therefore represents an increase in the concentration of allopregnanolone and a period of GABAergic plasticity[71]. Our observed differences in inhibitory connectivity in mothers at one-year postpartum may reflect the reorganisation of the GABAergic system at this time[30], providing a potential mechanism for our results. Plasticity of the GABAergic system across the peripartum is related to maternal behaviour, and dysregulation of GABAergic signalling in rodents results in deficits in maternal care, and increased symptoms of anxiety and depression[68]. Our interpretation of the selective modulation of inhibitory connectivity would be strengthened with the addition of hormonal data, which was not collected from this sample. Therefore, it is not possible to assert that the differences we observed are related to levels of allopregnanolone. Future research should investigate the relationships between fluctuating hormone levels and inhibitory connectivity across the peripartum period.

References

1 Friston, K. J., Kahan, J., Biswal, B. & Razi, A. A DCM for resting state fMRI. *Neuroimage* **94**, 396-407 (2014).

2 Voigt, K., Razi, A., Harding, I. H., Andrews, Z. B. & Verdejo-Garcia, A. Neural network modelling reveals changes in directional connectivity between cortical and hypothalamic regions with increased BMI. *International Journal of Obesity* **45**, 2447-2454 (2021).

3 Voigt, K., Murawski, C., Speer, S. & Bode, S. Effective brain connectivity at rest is associated with choice-induced preference formation. *Human brain mapping* **41**, 3077-3088 (2020).

4 Friston, K. *et al.* Bayesian model reduction and empirical Bayes for group (DCM) studies. *Neuroimage* **128**, 413-431 (2016).

5 Almeida, J. R. *et al.* Abnormally increased effective connectivity between parahippocampal gyrus and ventromedial prefrontal regions during emotion labeling in bipolar disorder. *Psychiatry Research: Neuroimaging* **174**, 195-201 (2009).

6 Vitale, E. M. & Smith, A. S. Neurobiology of Loneliness, Isolation, and Loss: Integrating Human and Animal Perspectives. *Frontiers in Behavioral Neuroscience*, 85 (2022).

7 Barba-Müller, E., Craddock, S., Carmona, S. & Hoekzema, E. Brain plasticity in pregnancy and the postpartum period: links to maternal caregiving and mental health. *Archives of Women's Mental Health* **22**, 289-299, doi:10.1007/s00737-018-0889-z (2018).

8 Hoekzema, E. *et al.* Pregnancy leads to long-lasting changes in human brain structure. *Nat Neurosci* **20**, 287-296, doi:10.1038/nn.4458 (2017).

9 Lisofsky, N. *et al.* Differences in navigation performance and postpartal striatal volume associated with pregnancy in humans. *Neurobiology of learning and memory* **134**, 400-407 (2016).

10 Hoekzema, E. *et al.* Becoming a mother entails anatomical changes in the ventral striatum of the human brain that facilitate its responsiveness to offspring cues. *Psychoneuroendocrinology* **112**, 104507, doi:10.1016/j.psyneuen.2019.104507 (2020).

11 Kim, P. *et al.* The plasticity of human maternal brain: longitudinal changes in brain anatomy during the early postpartum period. *Behav Neurosci* **124**, 695-700, doi:10.1037/a0020884 (2010).

12 Luders, E. *et al.* From baby brain to mommy brain: Widespread gray matter gain after giving birth. *Cortex* **126**, 334-342, doi:10.1016/j.cortex.2019.12.029 (2020).

13 Lisofsky, N., Gallinat, J., Lindenberger, U. & Kuhn, S. Postpartal Neural Plasticity of the Maternal Brain: Early Renormalization of Pregnancy-Related Decreases? *Neurosignals* **27**, 12-24, doi:10.33594/000000105 (2019).

14 Dufford, A. J., Erhart, A. & Kim, P. Maternal brain resting-state connectivity in the postpartum period. *J Neuroendocrinol* **31**, e12737, doi:10.1111/jne.12737 (2019).
15 Atzil, S., Hendler, T. & Feldman, R. Specifying the neurobiological basis of human attachment: brain, hormones, and behavior in synchronous and intrusive mothers. *Neuropsychopharmacology* **36**, 2603-2615, doi:10.1038/npp.2011.172 (2011).
16 Orchard, E. R. *et al.* Relationship between parenthood and cortical thickness in late adulthood. *PLOS ONE* **15**, e0236031, doi:10.1371/journal.pone.0236031 (2020).
17 Köhler, S. *et al.* Memory impairments associated with hippocampal versus parahippocampal-gyrus atrophy: an MR volumetry study in Alzheimer's disease. *Neuropsychologia* **36**, 901-914 (1998).
18 Burgmans, S. *et al.* The posterior parahippocampal gyrus is preferentially affected in age-related memory decline. *Neurobiology of aging* **32**, 1572-1578 (2011).
19 Davies, S. J., Lum, J. A., Skouteris, H., Byrne, L. K. & Hayden, M. J. Cognitive impairment during pregnancy: a meta-analysis. *Med J Aust* **208**, 35-40, doi:10.5694/mja17.00131 (2018).
20 Paul, S. *et al.* Neural pathways of maternal responding: systematic review and meta-analysis. *Archives of women's mental health* **22**, 179-187 (2019).
21 Chase, H. W., Moses-Kolko, E. L., Zevallos, C., Wisner, K. L. & Phillips, M. L. Disrupted posterior cingulate–amygdala connectivity in postpartum depressed women as measured with resting BOLD fMRI. *Social cognitive and affective neuroscience* **9**, 1069-1075 (2014).
22 Deligiannidis, K. M. *et al.* GABAergic neuroactive steroids and resting-state functional connectivity in postpartum depression: a preliminary study. *Journal of psychiatric research* **47**, 816-828 (2013).
23 MacKinnon, A. L. *et al.* The role of oxytocin in mothers' theory of mind and interactive behavior during the perinatal period. *Psychoneuroendocrinology* **48**, 52-63, doi:10.1016/j.psyneuen.2014.06.003 (2014).
24 Lenzi, D. *et al.* Neural basis of maternal communication and emotional expression processing during infant preverbal stage. *Cereb Cortex* **19**, 1124-1133, doi:10.1093/cercor/bhn153 (2009).
25 Pearson, R., Lightman, S. & Evans, J. Emotional sensitivity for motherhood: late pregnancy is associated with enhanced accuracy to encode emotional faces. *Hormones and behavior* **56**, 557-563 (2009).
26 Anderson, M. V. & Rutherford, M. Recognition of novel faces after single exposure is enhanced during pregnancy. *Evolutionary Psychology* **9**, 147470491100900107 (2011).
27 Golan, O., Baron-Cohen, S., Hill, J. J. & Golan, Y. The "reading the mind in films" task: complex emotion recognition in adults with and without autism spectrum conditions. *Social Neuroscience* **1**, 111-123 (2006).
28 Spreng*, R. N., McKinnon*, M. C., Mar, R. A. & Levine, B. The Toronto Empathy Questionnaire: Scale development and initial validation of a factor-analytic solution to multiple empathy measures. *Journal of personality assessment* **91**, 62-71 (2009).
